# Analysis of tire wear airstrip particles (TWAP)

**DOI:** 10.1038/s41598-022-19986-9

**Published:** 2022-09-23

**Authors:** Vanessa Spanheimer, Danka Katrakova-Krüger

**Affiliations:** TH Köln, 51643 Gummersbach, Germany

**Keywords:** Environmental sciences, Materials science

## Abstract

Tire wear is a main contributor to microplastics. As we cannot fully avoid tire wear, otherwise we could not brake and stop, new solutions are needed to address this problem. Not only on roads tire wear is released to the environment, even more can be found at airports. The advantage there is that the Tire Wear Airstrip Particles are gathered while cleaning the pavement. This collection is an opportunity to recycle and add new value to it. Whereas rubber powder is a common way to recycle and reuse end-of-life-tires as raw material in rubber compounds, the question is if TWAP is reusable in the same or similar way. In this study TWAP and rubber powder from truck tire treads are analyzed and compared with regard to their morphology, particle size distribution and composition. The particle size distribution of TWAP is broader than rubber powder containing also much smaller particles. The mineral content of TWAP is about 60%. These minerals can be residues of the pavement, brake wear but also rubber ingredients. In comparison to rubber powder, the impurities of TWAP are expected to have an impact with regard to potential applications and should be better separated.

## Introduction

Microplastic in the environment is a huge problem today and is expected to be even more significant in the future. One of the main contributors is tire wear. Tire Wear Road Particles (TWRP) account for a large proportion of microplastics in the environment. According to estimations from the Fraunhofer UMSICHT study, the emissions of tire wear particles into the environment amounts to nearly 100,000 tons per year in Germany alone^1^. In addition to wear from tires, particles from road markings, brakes and asphalt can also be found in wear samples^2,3^. The friction between the tire and the pavement during driving, speeding up and braking causes particles to be worn out from both, the tire and the pavement. Also, the brake pads are contributing to wear as the braking mechanism is to decelerate by friction. These particles are then carried out into the environment.

Tire wear does not exclusively come from passenger cars and trucks, but also from airplanes. There is no proper data regarding the contribution of airplane tires abrasion to this available. But Frankfurt Airport alone reported a volume of 83 tons of tire wear in 2019^4^. So, it can be assumed, that airports alone contribute more to tire wear particles than all passenger cars, busses and trucks together. Aircraft tires have to meet different requirements than passenger car or truck tires. There is more load, higher temperatures, higher acceleration and deceleration which lead to other properties of the Tire Wear Airstrip Particles (TWAP) in comparison to the TWRP on the highways.

The formation of wear is overall the same. Through friction between tire and pavement abrasion takes place which depends on the properties of the tire, the road surface, the vehicle (load and speed) and the driving behavior. This leads to heating and crack initiation and propagation until particles are worn off. Most studies on this have been conducted on car tires, both on the road and in laboratory simulations^2,5,6^. Kim and Lee produced and analyzed abrasion particles in a laboratory tire wear simulator under various conditions such as load, speed, and driving behavior. These tire wear particles had particle sizes smaller than 5 μm^7^. In contrast, tire abrasions from the environment have particle sizes up to 350 μm^8^. This can be due to the fact that they agglomerate fast after building. The particles produced by friction often take an elongated shape^7–9^. The chemical composition shows particularly high carbon and silicon contents as well as sulfur and heavy metals^7,10^. The presence of zinc in the abrasion particles, next to other organic compounds such as vulcanization accelerators, can be used as an indicator for better distinguishing them from other microplastic particles^11,12^. Abraded organic compounds and zinc can migrate out of the particles into the environment and further contribute to environmental pollution^2,13^.

However, since tire wear cannot be completely avoided, producers always try to optimize tires to generate less wear^14–17^ but in addition to the reduced emission, the recycling of the TWRP and TWAP should be considered. Therefore, the particles have to be gathered. On German roads the major part of wear is released to road banks and soils next to the roads. Furthermore, surface water which is gathered in wastewater treatment plants contains wear particles that are then becoming part of the sewage sludge which is used as agricultural fertilizer^18^. At airports the airstrips are cleaned up regularly for security reasons to maintain good grip properties for taking off and landing of the planes. This cleaning process takes place under high water pressure. After suction the water gets filtrated and a large part of the TWAP can be recovered as filter cake. Up to now these have only been recycled thermally, that means they are simply burnt. In order to come closer to the ideal of a circular economy, the use of TWAP would serve greatly as an additional source of secondary raw material, next to that of end-of-life-tires. The use of end-of-life-tires is already common and a great way to contribute to circular economy^19–21^. The tires are separated in steel, fibers and rubber. The rubber is then grinded to granulate or even finer rubber powder. The recycling of end-of-life-tires into rubber powder and granulate amounted to 251,000 tons in Germany in 2019^22^. Another recycling strategy is pyrolysis, used for the purposes of recovering oil, gas and carbon black^23^. This could also be applied for recycling TWAP.

To consider TWAP as a recycled material like the commonly used rubber powder, a comparison between those particles is essential to understand the challenges that will appear by using TWAP in new rubber compounds which will be the object of further studies. Up to now the process of formation of wear, the simulation of it and analysis to find and distinguish tire wear in different samples have been investigated. This study is focused on the comparison of TWAP to rubber powder and establishing TWAP as a recycling product instead of burning in order to give it a new value and contribute to circular economy.

## Experimental

The TWAP were made available by Volkmann Strassen- und Verkehrstechnik GmbH and originate from the airstrip cleaning of an airport in Germany. Larger contaminants such as leaves or sheet/packaging residues were removed beforehand. Additionally, the TWAP was dried to get rid of residual water from cleaning. For comparison, the rubber powder *Raumehl Type I* from Regupol BSW GmbH was used. This rubber powder is produced by ambient grinding and originates from truck tire treads. For comparison samples of a truck tire tread provided by Gummi Berger GmbH and an aircraft tire tread provided by the University of Twente were used for thermogravimetric analysis.

Microscopic images were taken using Olympus SZX10 stereomicroscope. The particle size distribution was measured with the Mastersizer 3000 from Malvern Panalytical. Both the rubber powder and the TWAP were first sieved with the AS 400 control planar sieve machine using a sieve with a 500 μm mesh size to exclude larger particles. Thermogravimetric analyses were carried out with the STA 409 PC from Netzsch, using the measuring parameters mentioned in Table 1. Additionally, the amount of mineral residues of the TWAP was determined by calcination as TWAP are very heterogeneous and sampling small amounts for TGA can lead to high variations in the results. With more specimen mass this effect can be eliminated. Five specimens around 5 g each of TWAP were taken and calcinated in the high temperature oven K1253A from Heraeus at a temperature of 900 °C according to the TGA. The elemental compositions of the starting products and the ash from TGA were subsequently analyzed by means of Energy-Dispersive X-Ray Spectroscopy (EDS) analysis with the scanning electron microscope SU5000 from Hitachi and EDS sensor X-Max 80 from Oxford Instruments. Elemental analysis with EDS is suitable to detect elements qualitatively but not quantitatively. With the elemental analyzer for carbon (C), nitrogen (N), hydrogen (H) and sulfur (S) Vario Macro Cube from Elementar the percentual amount of these elements was measured in TWAP and rubber powder. In contrast to EDS, the CNHS-analysis can measure the light weight elements hydrogen and nitrogen and additionally measures the four elements quantitatively. As organic materials like rubber consist mainly of carbon and hydrogen, this method is suitable to complete the analysis of the whole elemental spectrum. With the quantitative measurement it is possible to reveal the composition of TWAP and rubber powder and estimate the amount of pure rubber particles within TWAP. Polycyclic aromatic hydrocarbon (PAH) analysis was examined in accordance to AfPS GS 2019:01—PAK by TÜV Rheinland LGA Products GmbH. This method measures the amount of 15 PAHs and is used for issuing products with the GS-Mark. The samples are extracted with toluene for 1 h at 60 °C in an ultrasonic bath, cooled to room temperature, purified and measured with a gas chromatograph with mass-specific detector.

**Table 1:** Measuring parameters TGA.

Mode	Temperature (°C)	Heating rate	Gas
Start	30C		N_2_ (30 ml/min)
Dynamic	600	10 K/min	N_2_ (30 ml/min)
Dynamic	900	10 K/min	N_2_, O_2_ (20 + 10 ml/min)

## Results and discussion

### Particle size distribution

**Figure 1:**
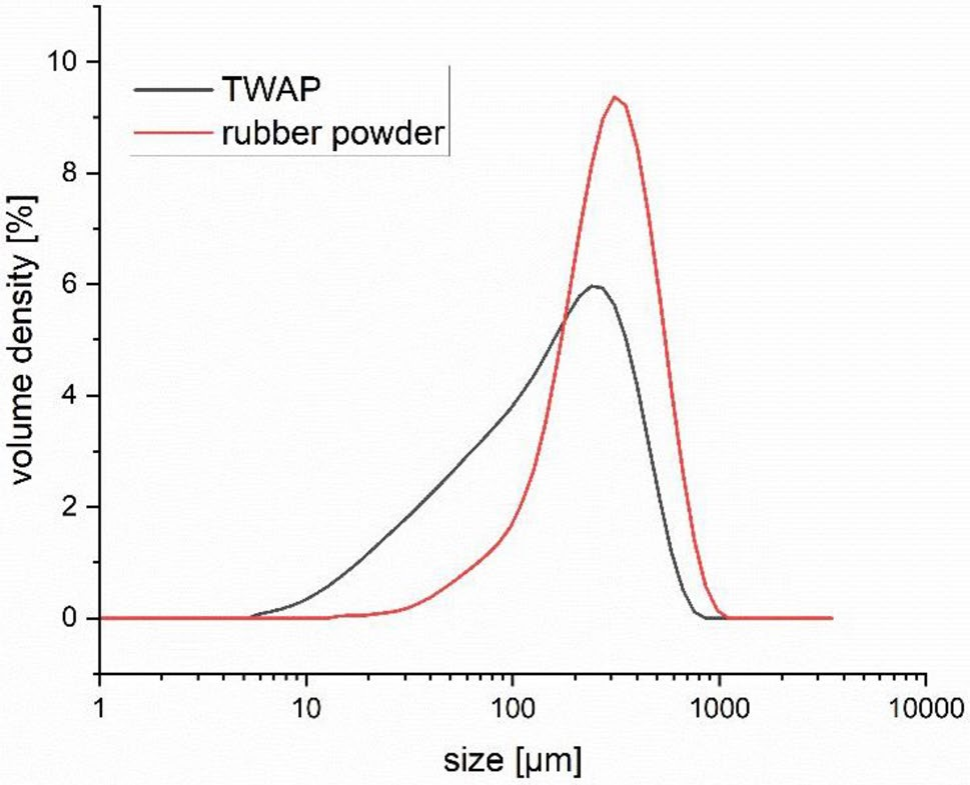
Particle size distribution of rubber powder and TWAP.

Despite the sieving process, large particles can still be found in both the TWAP and the rubber powder. The TWAP showing particle sizes of up to 750 μm and the rubber powder even up to 1 mm (see Fig. 1). Reagglomeration after sieving and elongated particles which pass the mesh with their smaller width are leading to higher particle sizes. The particle size distribution of TWAP is significantly wider than that of the rubber powder and also exhibits much smaller particles. The abrasion process produces finer particles of the different materials than the grinding of rubber. This is favorable for the use of TWAP as a secondary raw material similar to rubber powder in rubber compounds.

### Calcination

Calcination of the TWAP yields a quantity of approx. 62% mineral constituents. This agrees well with the observations made by TGA (s. 4.3) and the chemical composition by elemental analysis (s. 4.5). Further information with regard to the composition of the TWAP and the rubber powder is given by the TGA.

### Thermogravimetric analysis

**Figure 2:**
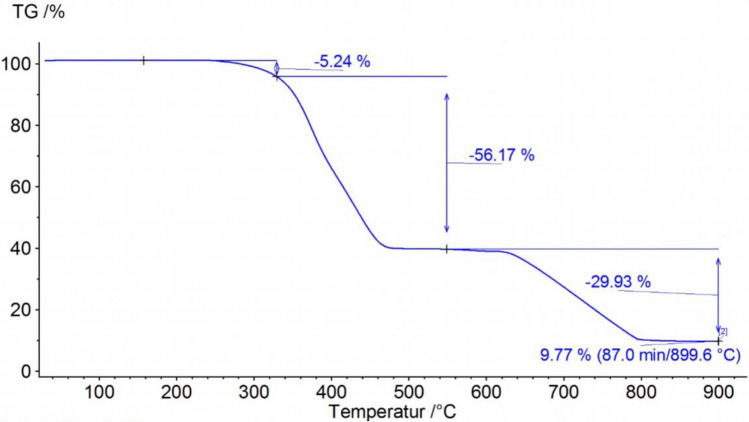
Thermogravimetric analysis of rubber powder.

The thermogravimetric analysis of rubber powder is shown in Fig. 2. From room temperature to approximately 330 °C a mass loss of 5.24% can be seen. This is related to volatile matter, softener and other low-molecular additives. The next big mass change of 56.17% from 330 to 480 °C is due to degradation of polymers. The degradation temperatures of natural rubber and butadiene rubber, which are used typically in truck treads, lie around 370 °C to 480 °C. At 600 °C the atmosphere is changed from pure nitrogen to nitrogen and oxygen. From this point on Carbon Black as well as other residuals consisting of carbon are oxidizing and leaving the specimen as CO_2_ gas. This results in a further mass change of 29.93%. A residual mass of 9.77% is obtained which consists of minerals. The composition of the ash will be shown in the elemental analysis (s. 4.5).

**Figure 3:**
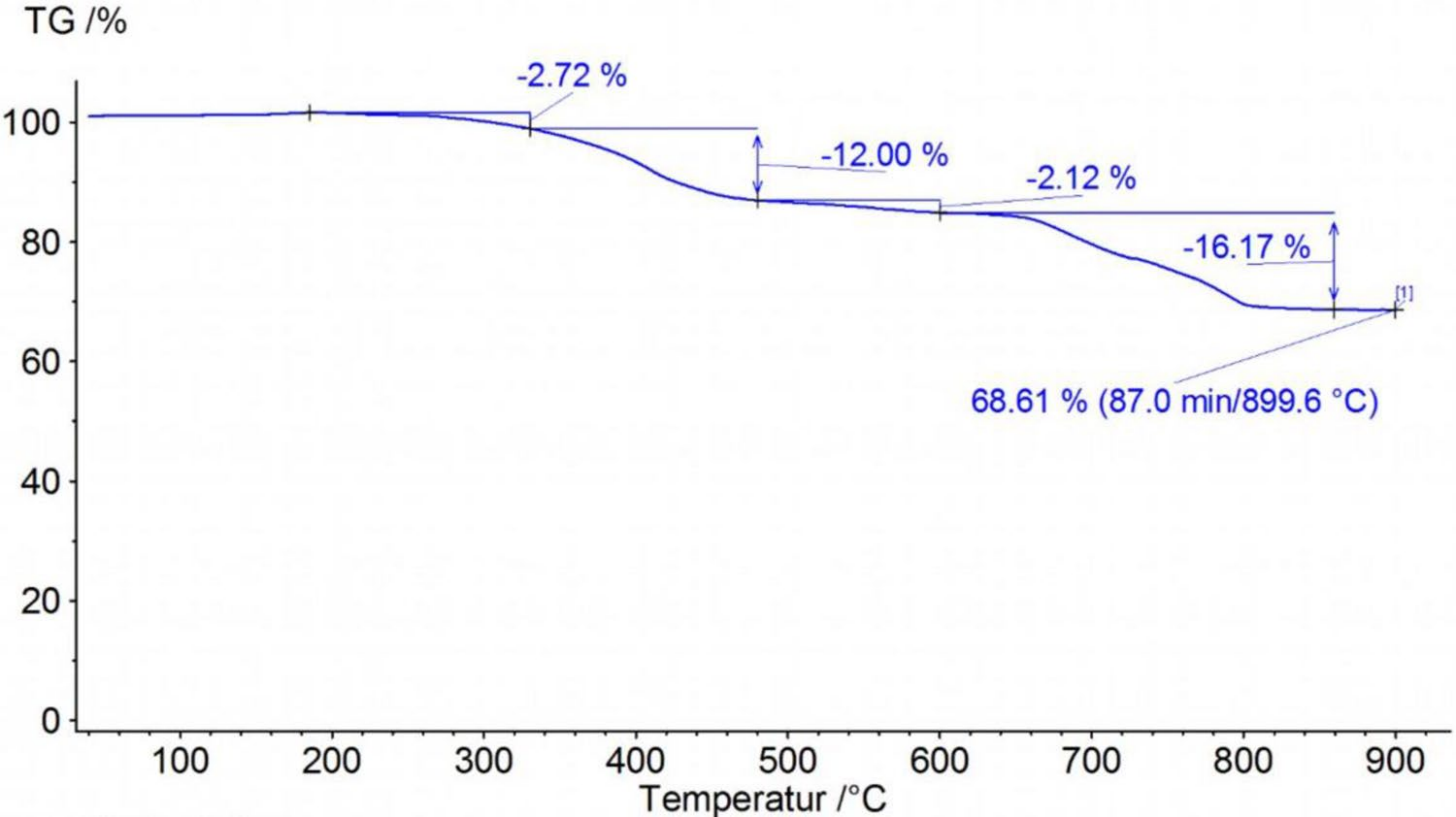
Thermogravimetric analysis of TWAP.

Figure 3 shows the thermogravimetric analysis of TWAP. In the first range up to 330 °C a mass loss of 2.72% can be seen. Again, this can be related to volatile and low-molecular matter. In contrast to the TGA of rubber powder the graph of TWAP shows no clear change in degradation rate going from the degradation temperature range of volatile matter to the range of polymers. A mass loss of 12% up to 480 °C is seen and a further 2.12% up to 600 °C. Both can be related to polymers. While rubber powder consists mainly of NR and BR, a mixture of more polymers can be expected for TWAP. These other polymers can origin from road markings and asphalt. Again, the change of atmosphere leads to oxidation of carbon which leads to a mass loss of 16.17% for the TWAP. A residual mass of 68.61% is obtained.

**Figure 4:**
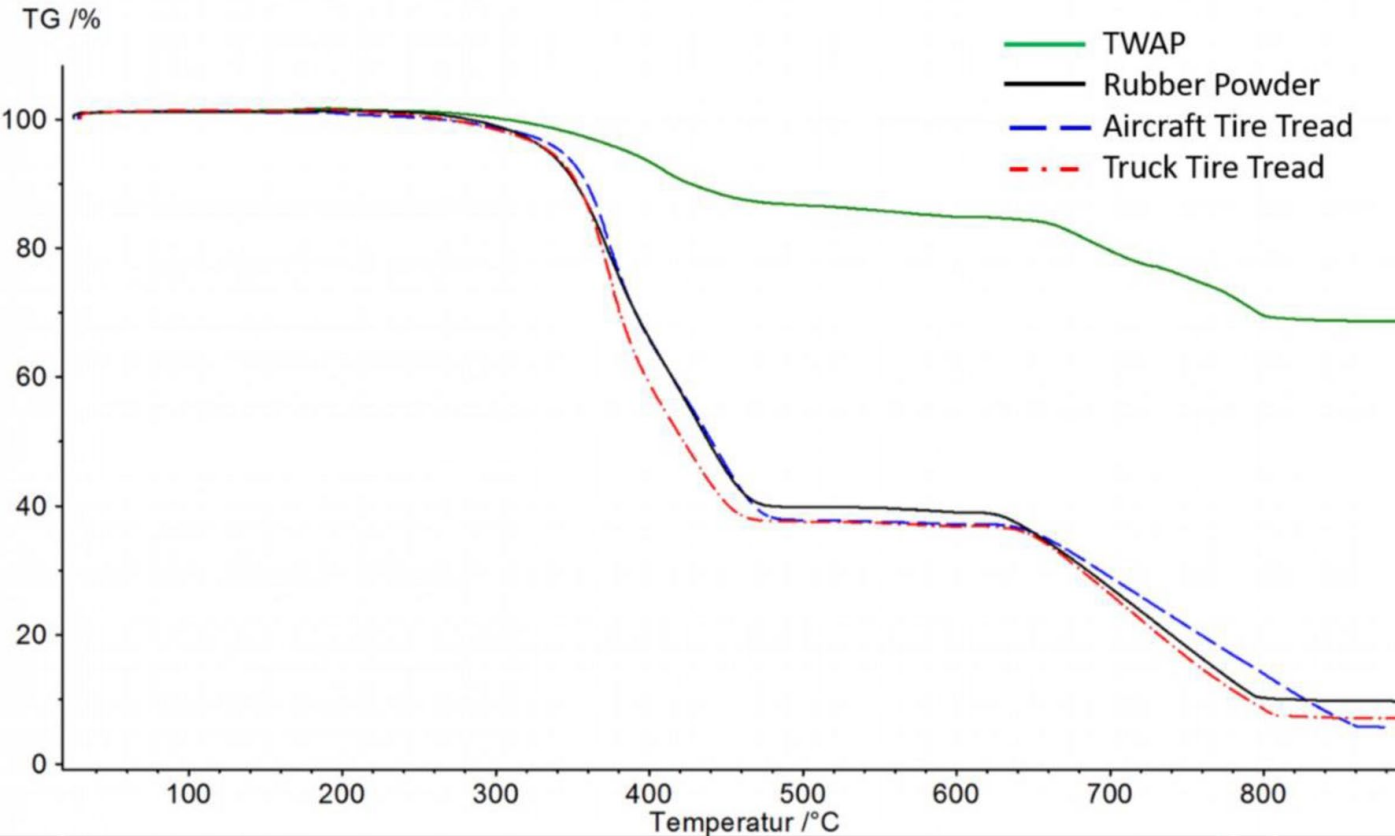
Comparison of TGA-Analyses for TWAP, rubber powder, aircraft and truck tire tread.

To compare TWAP and rubber powder with their origin from tires, TGA-analyses of a truck tire tread respectively an aircraft tire tread were examined (see Fig. 4). The results of rubber powder, aircraft tire tread and truck tire tread are similar. Differences of them can be derived from varying compound recipes of different tire producers. As TWAP contain a high amount of mineral residues, the mass changes for all TGA-measurements where normalized. The sum of all mass changes was set as 100% in order to eliminate the influence of the residual mass. The results of the normalized mass changes can be seen in Table 2. Again, the rubber powder and truck tire tread are very similar. The aircraft tire tread reveals a significantly higher portion of volatile and low-molecular matter as well as lower polymer content than rubber powder and truck tire tread. The amounts of Carbon Black are comparable.

**Table 2:** Normalized mass changes of thermogravimetric analyses.

	Rubber powder (%)	Truck tire tread (%)	TWAP (%)	Aircraft tire tread (%)
Up to 330 °C	5.74	5.73	8.24	9.15
Up to 600 °C	61.50	62.19	42.77	57.39
Up to 900 °C	32.77	32.08	48.99	33.46

In contrast, TWAP show a mass loss of volatile and low-molecular matter nearly like the aircraft tire tread. But a difference of 15–20% less polymer content in comparison to the aircraft tire tread respectively rubber powder/truck tire tread can be seen. Also, TWAP have nearly 50% carbon content which is much higher than in the aircraft tire tread. This may be due to additional organic matter coming from the pavement abrasion that contains bitumen.

### Light microscopy and SEM

**Figure 5:**
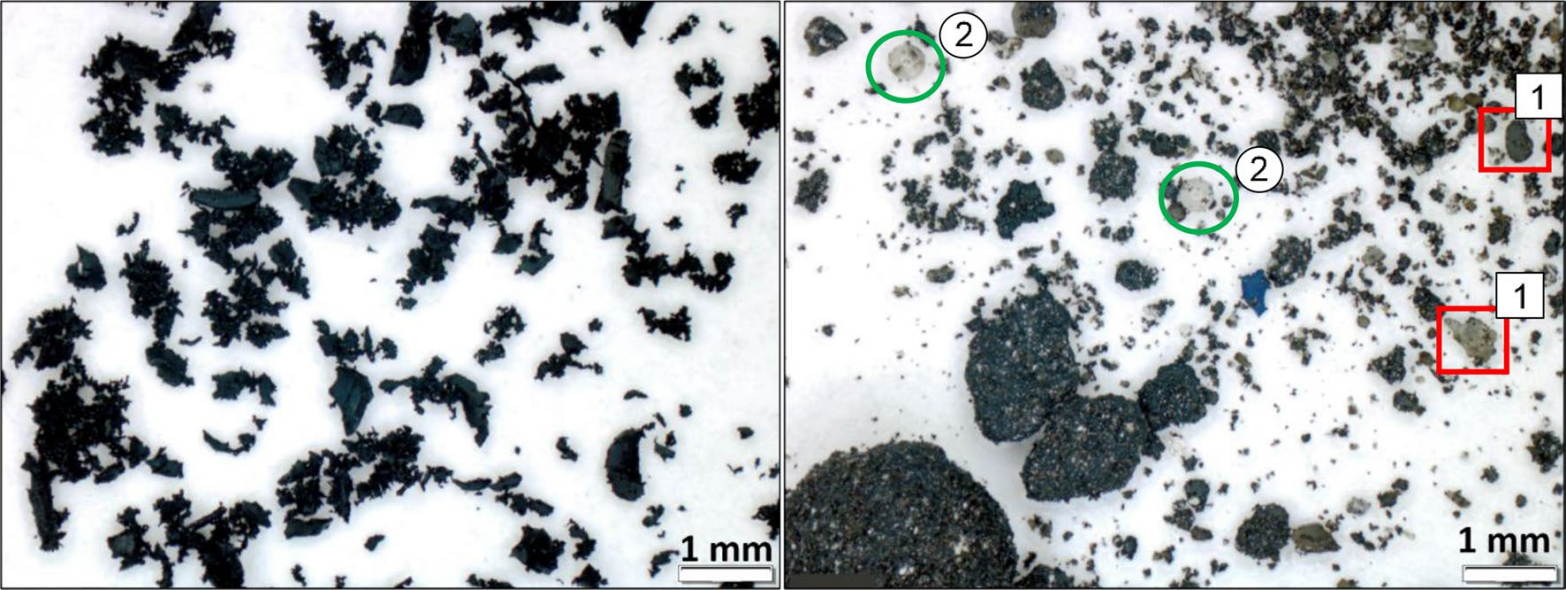
Microscopic image of rubber powder (left) and TWAP (right).

The microscopic image shows that the rubber powder is free from other particles. The particles have different sizes and morphology. The rubber powder has a rough, jagged surface (see Fig. 5 left) which originates from the ambient grinding process. Here, the grinding is done at room temperature, where the rubber is soft and elastic.

**Figure 6:**
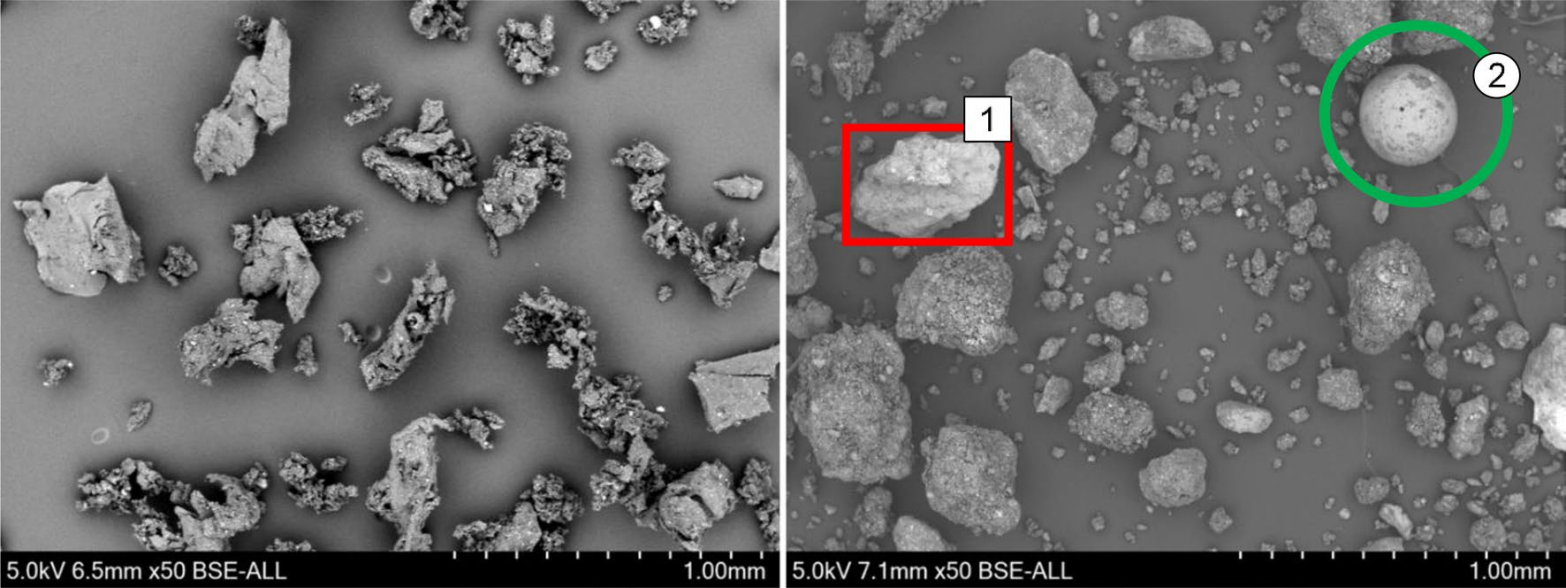
SEM image (50x) of rubber powder (left) and TWAP (right).

The TWAP, on the other hand, consists of particles of much more different sizes and composition (see Fig. 5 right). The dark particles come mainly from aircraft tire abrasion agglomerated with other particles. During landing, driving and braking, particles are released from the tread due to friction on the pavement. Here, temperature can come close to 200 °C^24^. Sand grains (1) and glass beads (2) are found. Sand grains may originate from surrounding areas of the pavement. The glass beads are used in road markings to reflect the light for a better visibility. In the backscattered electrons (BSE) image they differ in brightness due to the different chemical composition (see Fig. 6).

**Figure 7:**
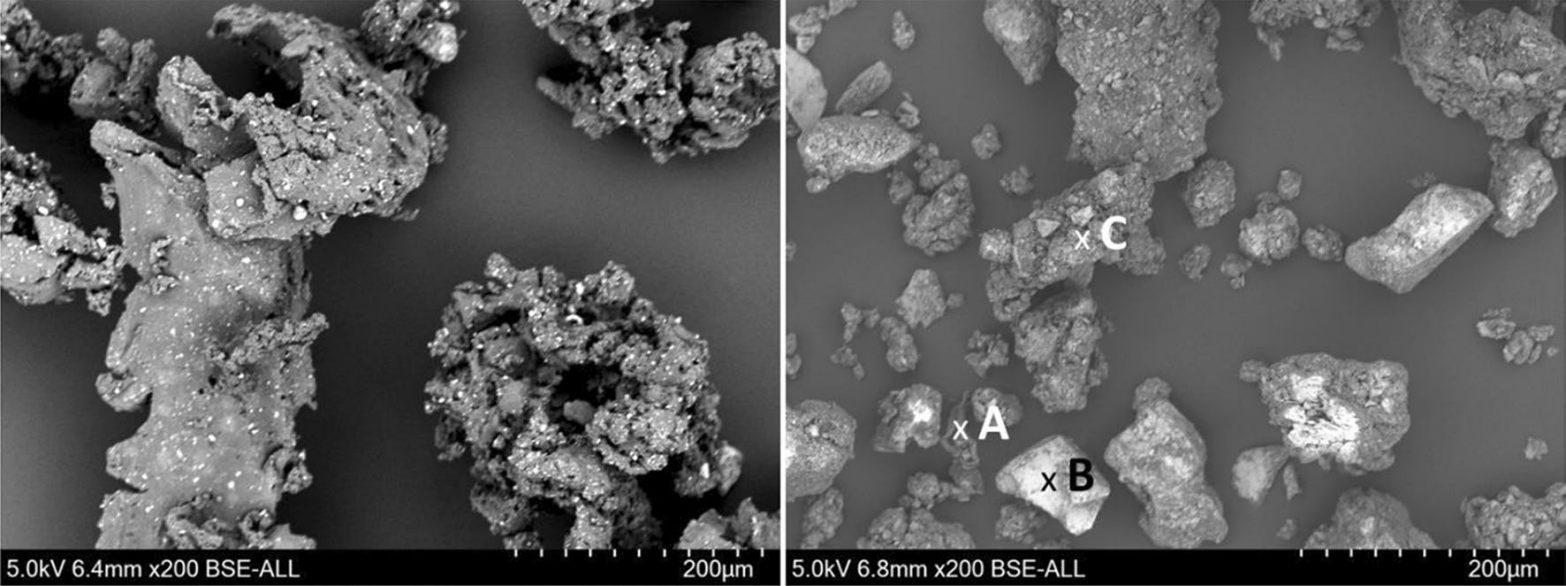
SEM image of rubber powder (left) and TWAP (right) at higher magnification (200x).

The SEM images of rubber powder and TWAP also show the different particle sizes. The morphology is clearer in these images. The ambient grinding process of rubber powder from a tire leads to particles with a rough surface (see Figs. 6 and 7). On the surface of the rubber powder are some small particles found which reveal a higher density (brighter color in SEM). TWAP show some particles with a smooth surface like the glass beads (2) and some mineral particles (1, B) (see Figs. 6 and 7). The agglomerates of tire, break and pavement wear (C) are forming bigger particles (see Fig. 7). Rubber from tire wear alone is rarely found in the TWAP (A). The rubber particles show an elongated shape and are smaller than rubber powder particles obtained by grinding. The abrasion process takes place under higher temperatures and loadings than ambient grinding. Due to their small size most particles form directly after abrasion agglomerates with other wear particles which adhere strongly.

### Elemental analysis

EDS-Measurements taken before and after the TGA measurements of TWAP and rubber powder show the elemental compositions of the starting materials and the remaining ashes.

**Figure 8:**
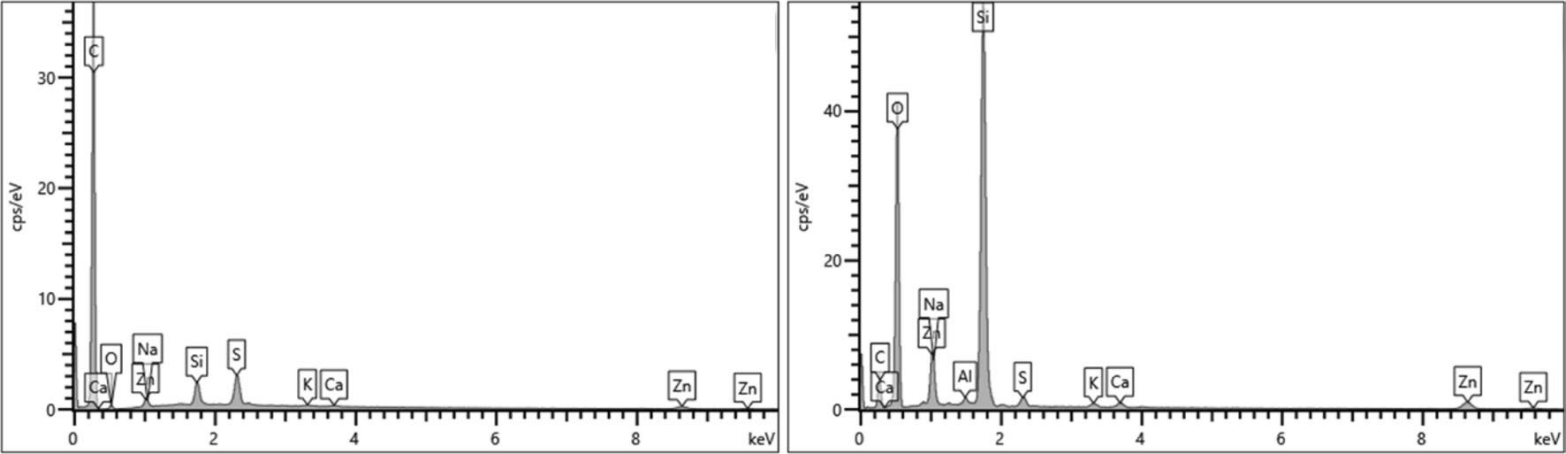
Elemental spectra of rubber powder before (left) and after (right) calcination.

The elemental analysis shows a very high carbon content (C) for the rubber powder. This is logical for a rubber containing Carbon Black (pure carbon) as a reinforcing filler, polymers and plasticizers (consisting of hydrocarbons). Zinc (Zn) as zinc oxide is a typical ingredient in rubber compounds for activating the vulcanization process. In addition, the crosslinking agent sulfur (S) is also present. Traces of silicon (Si), calcium (Ca), sodium (Na) and potassium (K) are found (see Fig. 8 left). The mineral residue obtained after calcination consists mainly of silicon and oxygen. These may originate from the pavement and road dust that adheres to the tread. Again, the rubber compound ingredients sulfur and zinc as zinc oxide are found. (see Fig. 8 right). The elements calcium, sodium, potassium and aluminum are detected in small amounts and may originate from break wear^25–28^.

**Figure 9:**
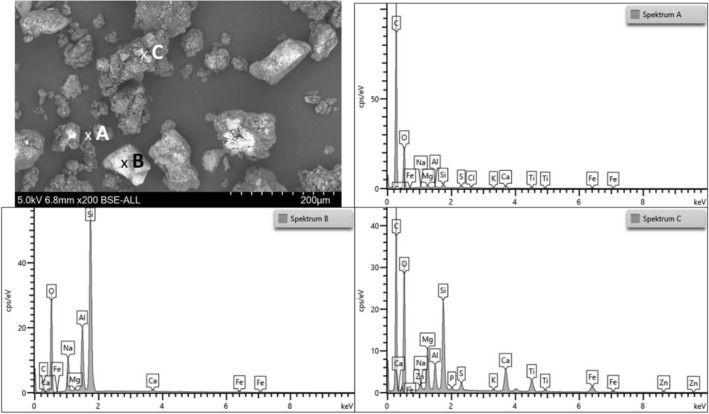
Elemental spectra of TWAP: (**A**) rubber particle, (**B**) mineral particle and (**C**) agglomerate of different particles.

Much like the spectrum of the rubber powder, the elemental analysis spectrum A of the TWAP shows a high carbon (C) content as well. In addition to sulfur (S), silicon (Si), oxygen (O), sodium (Na) and calcium (Ca), more aluminum (Al) and other metals such as iron (Fe), magnesium (Mg), potassium (K) and titanium (Ti) are also found (see Fig. 9). These may originate from silicon oxide (SiO_2_) as reinforcing filler in rubber compounds and brake wear that adhere to the rubber particles. Brake pads contain a huge variety of different materials^25–28^. Traces of chlorine (Cl) are found, which may come from sodium chloride used for deicing the pavement^20^.

Spectrum B shows the elemental composition of a mineral particle. The main elements are silicon (Si) and oxygen (O). This indicates that the particle consists mainly of silicon oxide like a sand grain. Aluminum (Al), iron (Fe), calcium (Ca) and sodium (Na) are found (see Fig. 9). These are elements used in brake pads. Some brake wear particles may be agglomerated to the bigger particle. Carbon (C) can originate from diverse organic resources.

The elemental spectrum C) shows the composition of an agglomerate of different particles. Carbon (C), silicon (Si) and oxygen (O) are the main elements found which is consistent with spectra A) and B). Zinc (Zn) from the vulcanization activator and sulfur (S) as crosslinking agent are found. Again, aluminum (Al), iron (Fe) calcium (Ca), sodium (Na), magnesium (Mg), potassium (K) and titanium (Ti) indicate the presence of break wear particles. Traces of phosphorus (P) are measured (see Fig. 9). Phosphorus is used as phosphate in fertilizer and may originate from soil dust^29^.

**Figure 10:**
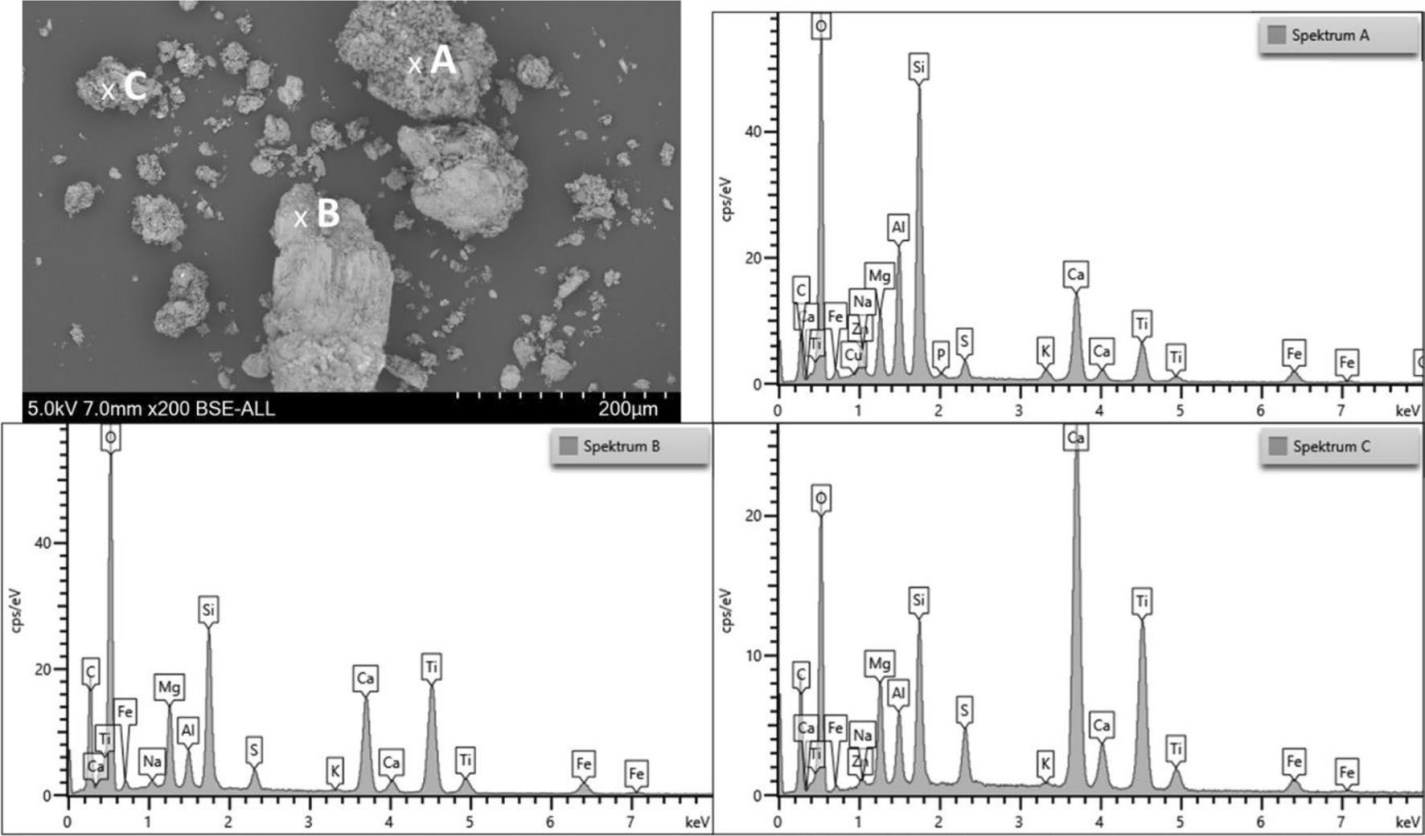
Elemental spectra of TWAP after calcination on different particles.

After calcination, silicon (Si) and oxygen (O) are still the most common elements found. These originate not only from the silica in the rubber particles, but also from sand and glass beads used in road markings (see Fig. 10). As seen in the spectra before calcium (Ca), titanium (Ti), magnesium (Mg), iron (Fe), sodium (Na), magnesium (Mg), aluminum (Al), potassium (K) and manganese (Mn) is found which may originate from brake pads.

As the previous elemental analyses cannot give quantitative values and is not able to measure light elements, the CNHS-analysis was used complementary to the previous results. Rubber powder reveals approximately 90% of carbon and hydrogen (see Table 3) which is reasonable because it consists of pure rubber filled with Carbon Black. This is consistent with the mass loss in TGA of volatile matter, polymers and Carbon Black. The low amounts of nitrogen can be part of vulcanization accelerators, the sulfur as mentioned before is used as curing agent. All other elements have a quantity of about 8%. This summed up with the amount of sulfur agrees well with the measured residual mass in the TGA.

**Table 3:** Weight percentage of C, N, H and S in rubber powder and TWAP.

	C (%)	N (%)	H (%)	S (%)	Other (%)
Rubber powder	82.39	0.42	8.19	1.29	7.73
TWAP	26.47	0.21	2.79	0.61	69.95

The amounts of C, N, H and S in TWAP differ significantly from rubber powder. It contains only about 30% C and H, this is also in agreement with TGA-measurement. The low amount of nitrogen may also come from vulcanization accelerators and the sulfur from the curing agent. Other elements sum up to 70%, which presumably consist mainly of silicon and oxygen as seen in SEM/EDS analyses. This amount is also consistent with the residual mass and calcination results.

### PAH analysis

The European Union regulation REACH (Registration, Evaluation, Authorization and Restriction of Chemicals) set maximum amounts of polycyclic aromatic hydrocarbons (PAHs) for different products^30^. PAHs are seen as potential toxic substances and therefore REACH limits their amounts in products to reduce the health risks of consumers. As the further aim of this study is to reuse TWAP in new rubber products, the amount of PAHs is one important factor for consideration. As the filler carbon black and softener oil commonly used in rubber compounds are containing PAH, there is a maximum amount of additional PAH which can be accepted for other raw materials to hold the limit of REACH.

TWAP exhibit significantly less PAHs than rubber powder (see Table 4). This is an important advantage of TWAP versus rubber powder with regard to their use as a secondary raw material. One possible reason is that TWAP contain over 60% mineral residues and reveals significantly less C- and H-content than rubber powder. But as the difference is more than these amounts can explain, there are more influencing factors. It is possible that the softeners and other low volatiles decompose at the higher temperatures observed in the airplane tire treads at take off and landing. They were mentioned as one of the big contributors to PAHs in tires.

**Table 4:** PAH amounts of TWAP and rubber powder measured according to AfPS GS 2019:01 PAK.

	TWAP mg/kg	Rubber powder mg/kg
Phenanthrene	0.2	4
Pyrene	0.7	14
Anthracene	< 0.2	0.4
Fluoranthene	0.3	5.4
Naphthalene	< 0.2	1
Benzo(a)pyrene	< 0.2	0.5
Benzo(e)pyrene	< 0.2	1.2
Benz(a)anthracene	< 0.2	< 0.2
Benzo(b)fluoranthene	< 0.2	0.3
Benzo(j)fluoranthene	< 0.2	< 0.2
Benzo(k)fluoranthene	< 0.2	< 0.2
Chrysene	< 0.2	0.3
Dibenz(ah)anthracene	< 0.2	< 0.2
Benzo(ghi)perylene	0.5	2.7
Indene (1,2,3-cd)pyrene	< 0.2	0.2
**Sum 15 PAH**	**1.7**	**30.0**

## Conclusion and outlook

In this study TWAP was compared to rubber powder with regard to its suitability as secondary raw material in rubber compounds. TWAP show a broader particle size distribution than rubber powder and much more finer particles which is an advantage for using it in new rubber compounds. The calcination of TWAP as well as TGA and CHNS analyses reveal a mineral residue of approximately 60%. This mineral residue originates from sand grains and glass beads which are found in microscopic and SEM images, from brake wear and to a lesser extent from some inorganic rubber ingredients. The tire particles that were found in the TWAP are much smaller than rubber powder particles but are morphologically similar and show an elongated shape. TWAP mostly form agglomerates from tire, pavement and brake wear. The morphology and chemical composition of the TWAP are consistent with the findings of other studies regarding TWRP^7–10^. With regard to use TWAP as a secondary raw material, separation—especially of the bigger particles (sand, glass beads)—might be favorable. The TGA show, that TWAP have a polymer content about 15–20% which is lower than the polymer content of tires. Normally, truck and aircraft tire treads contain around 30% carbon black as filler. The measured content of carbon in TWAP is nearly 50% which comes from other organic matter like bitumen from the pavement wear. PAH analysis reveals significantly less PAHs for TWAP which facilitates the use in new products with regard to REACH. TWAP contains only 1.7 mg/kg PAH while rubber powder contains 30 mg/kg. It is shown, that rubber powder and TWAP have some properties in common, some like particle size and PAH content of TWAP are even better than rubber powder in regard to using it as a recycling material in rubber compounds. As such, the next step is to use TWAP in rubber compounds in the same way rubber powder is used today. This will allow to investigate the influence of the adherent metallic and other constituents on the compound properties. In case they influence negatively mechanical and/or chemical methods for separation could be considered.

## Data Availability

The dataset generated during and/or analyzed during the current study are available from the corresponding author on reasonable request.
